# Biosensing firefly luciferin synthesis in bacteria reveals a cysteine-dependent quinone detoxification route in Coleoptera

**DOI:** 10.1038/s41598-022-17205-z

**Published:** 2022-08-31

**Authors:** Daniel Rangel de Souza, Jaqueline Rodrigues Silva, Ariele Moreira, Vadim R. Viviani

**Affiliations:** 1 Graduate Program of Biotechnology and Environmental Monitoring, Federal University of São Carlos, Sorocaba, Brazil; 2 Departament of Physics, Chemistry and Mathematics, Federal University of São Carlos, Sorocaba, Brazil

**Keywords:** Biochemistry, Cell biology, Evolution

## Abstract

Luciferin biosynthetic origin and alternative biological functions during the evolution of beetles remain unknown. We have set up a bioluminescent sensing method for luciferin synthesis from cysteine and benzoquinone using *E. coli* and *Pichia pastoris* expressing the bright *Amydetes vivianii* firefly and *P. termitilluminans* click beetle luciferases. In the presence of d-cysteine and benzoquinone, intense bioluminescence is quickly produced, indicating the expected formation of d-luciferin. Starting with l-cysteine and benzoquinone, the bioluminescence is weaker and delayed, indicating that bacteria produce l-luciferin, and then racemize it to d-luciferin in the presence of endogenous esterases, CoA and luciferase. In bacteria the *p*-benzoquinone toxicity (I_C50_ ~ 25 µM) is considerably reduced in the presence of cysteine, maintaining cell viability at 3.6 mM *p*-benzoquinone concomitantly with the formation of luciferin. Transcriptional analysis showed the presence of gene products involved with the sclerotization/tanning in the photogenic tissues, suggesting a possible link between these pathways and bioluminescence. The lack of two enzymes involved with the last steps of these pathways, indicate the possible accumulation of toxic quinone intermediates in the lanterns. These results and the abundance of cysteine producing enzymes suggest that luciferin first appeared as a detoxification byproduct of cysteine reaction with accumulated toxic quinone intermediates during the evolution of sclerotization/tanning in Coleoptera.

## Introduction

Bioluminescence, the emission of visible light by living organisms arose many times during evolution^1^. Bioluminescence is originated by oxidative reactions in which compounds called luciferins are oxidized by molecular oxygen in the presence of enzymes called luciferases^2^. The biochemistry of some bioluminescent organisms including bacteria, dinoflagellates, coelenterates, crustaceans and beetles is already quite well known^3^. However, the origin of bioluminescent systems remains largely unknown.

In fireflies and other related beetles, bioluminescence is generated by a reaction involving a benzothiazolic luciferin, MgATP, O_2_ and 60 kDa luciferases which likely evolved from enzymes of the superfamily of CoA-ligases^4^. On the other hand, the biosynthetic pathway of luciferin and its original biological function in beetles still remain to be elucidated.

The chemical composition and properties of firefly luciferin have been investigated since 1950^5,6^. The chemical structure and synthesis were reported by White et al. in 1961. The reaction of 2-cyano-6-hydroxybenzothiazole with d-cysteine is the final step of this synthesis^7,8^. Isotope labelling of cysteine indicated that cysteine and benzoquinones are the likely metabolic precursors of luciferin in fireflies^9,10^. More recently, Kanie et al. demonstrated that luciferin can be spontaneously formed from cysteine (Cys) and *p*-benzoquinones (BQ) in a neutral buffered solution without the presence of enzymes or cofactors^11^. The simplicity of this spontaneous process can bring interesting clues about the natural and evolutionary origin of this substrate in fireflies.

Niwa et al. proposed that in firefly larvae and pupae, l-luciferin could be first synthesized from the more abundant l-cysteine, and then enzymatically converted to d-luciferin in the presence of ATP, Mg^2+^, CoA and luciferase in presence of an esterase^12^. Later, researchers from the University of Nagoya have shown that luciferin biosynthesis in the adult lanterns of the firefly species *Luciola lateralis* occurs from the reaction of l-cysteine with *p*-benzoquinone, or 1,4-hydroquinone, followed by decarboxylation of l-cysteine^13^.

Studies on luciferin biosynthesis in fireflies using the labeled 2-*S*-cystenylhydroquinone (Cys-HQ) isotope indicated that firefly luciferin can also be biosynthesized upon the incorporation of this molecule. Comparison the rate of incorporation of Cys-HQ at different stages of the life cycle suggests that luciferin biosynthesis occurs predominantly during the pupal stage of these insects^14^.

Strause et al., studying the biochemical and morphological changes during the development of lanterns in different life stages of the firefly species *Photuris pennsylvanica*, observed that the rate of luciferin synthesis is not the same at all stages of the metamorphosis, being more abundant in the larval and pupal stages^15^.

Recently, attempts to elucidate the biosynthetic pathway of beetle luciferin also used transcriptional studies of firefly lanterns^11,13,15–20^, and comparatively, both photogenic and non-photogenic tissues of fireflies, click beetles and railroad worms, and non-luminescent Elateroidea^18,19^. These studies highlighted the occurrence of active cysteine metabolism as well as abundance of phenoloxidases in firefly lanterns^16,18^. Additionally, the transcriptomic studies from firefly lanterns also showed transcripts related to pigmentation/sclerotization in this tissue^16,18,19^. However, the participation of these gene products in such biosynthetic pathway has not been confirmed by experimental studies, yet.

Departing from the luciferin spontaneous synthesis protocol of luciferin from *p*-benzoquinone and cysteine, reported by Kanie et al., and *E. coli* bacteria transformed with the bright and stable *Amydetes vivianii* firefly luciferase, we have set up a cellular bioluminescent assay for luciferin synthesis. Using this assay we showed that, as expected, benzoquinone is extremely toxic to bacteria, but in the presence of cysteine, this toxicity is dramatically reduced, resulting in the concomitant formation of luciferin. The participation of laccase in the luciferin biosynthetic pathway from hydroquinone was also confirmed. The above results and transcriptional analysis indicate the existence of a link between the sclerotization and tanning pathways with luciferin biossynthesis in beetles.

## Results and discussion

### Spontaneous synthesis of luciferin

Previously, Kanie et al., demonstrated that firefly luciferin can be spontaneously produced from *p*-benzoquinone and d-cysteine in buffered solution at neutral pH. We reproduced these results, but in the additional presence of the bright *Amydetes vivianii* firefly luciferase (AmyLuc) and MgATP, to sense the luciferin synthesis by bioluminescence detection using CCD camera system. We performed the assays using d-cysteine and its l-enantiomer. The results showed that, as expected, only the luciferin formed departing the d-cysteine produced bioluminescence.

**Figure 1:**
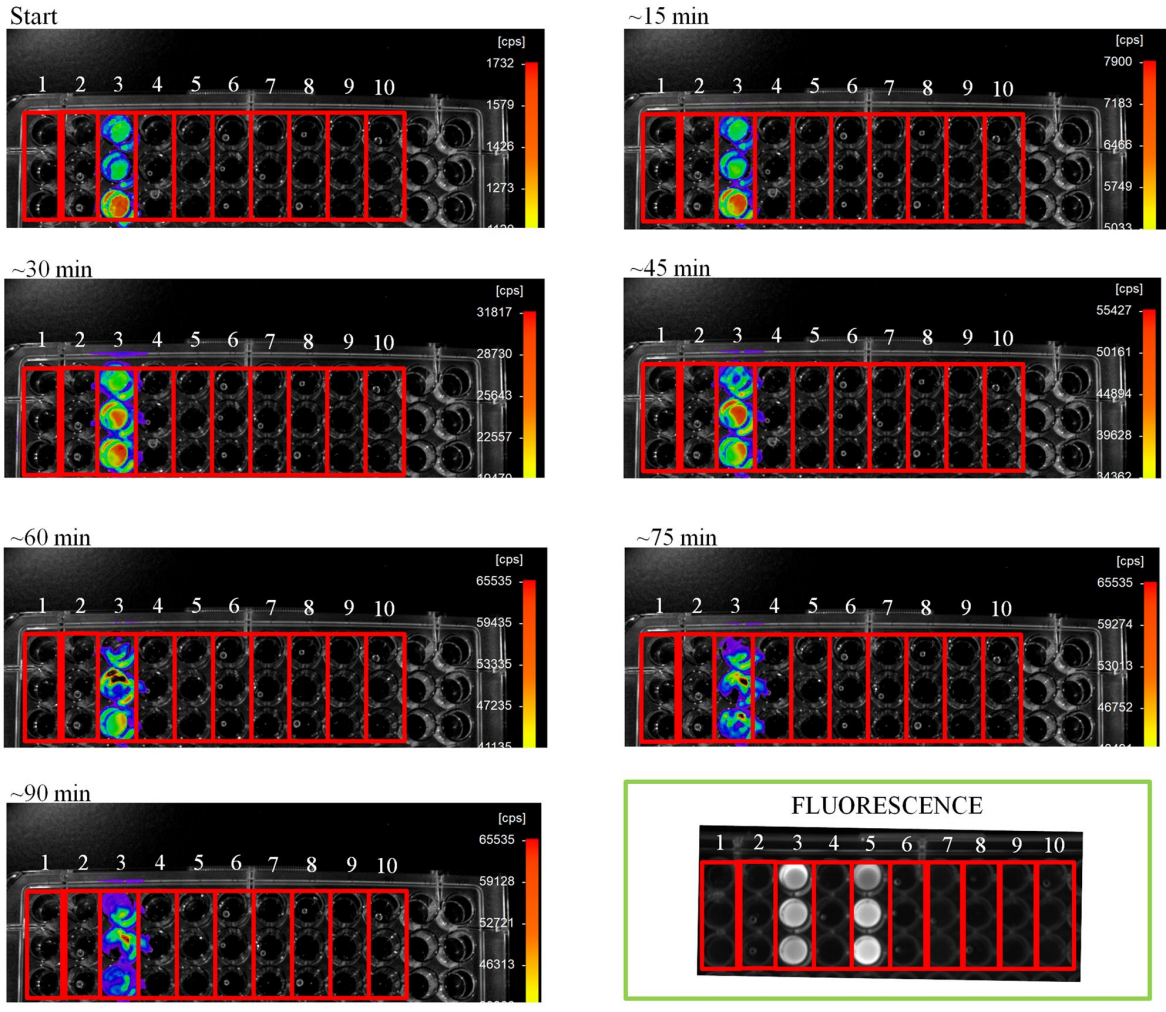
(Panels 0 to ~ 90 min) CCD bioluminescence detection of in vitro luciferin formation from d-cystein and *p*-benzoquinone (wells 3). (Panel “FLUORESCENCE”) fluorescence of luciferin upon UV irradiation also indicates luciferin formation from d-cysteine and *p*-benzoquinone in well 3, and from l-cysteine and *p*-benzoquinone in well 5. (1) Buffer + MgATP; (2) Buffer + MgATP + AmyLuc; (3) Buffer + MgATP + AmyLuc + d-cys + BQ; (4) Buffer + MgATP + AmyLuc + d-Cys; (5) Buffer + MgATP + AmyLuc + l-cys + BQ; (6) Buffer + MgATP + AmyLuc + l-cys; (7) Buffer + MgATP + AmyLuc + l-cys; (8) Buffer + MgATP + AmyLuc + d-cys + Hydroquinone; (9) Buffer + MgATP + AmyLuc + l-cys + Hydroquinone; (10) Buffer + MgATP + AmyLuc + Hydroquinone.

CCD imaging (Fig. 1) showed that bioluminescence activity occurred only in the reaction well 3, with d-cysteine and *p*-benzoquinone, confirming d-luciferin formation. Upon UV light irradiation (panel fluorescence, Fig. 1), the wells 3 and 5, which contained the reaction of *p*-benzoquinone with d-cysteine and l-cysteine, respectively, also displayed fluorescence, which is indicative of luciferin formation. Both l-luciferin and d-luciferin are fluorescent, however only d-luciferin participates in the bioluminescent reaction with luciferase, O_2_, and MgATP.

### Participation of hydroquinone, dopamine and laccase in the synthesis of luciferin

As already well demonstrated, luciferin can be biosynthesized from cysteine and benzoquinone^11,13^. In beetles, benzoquinones and other quinones are abundantly found in the tanning and sclerotization pathways. The immediate precursors of benzoquinone are hydroquinone and DOPA, which can be oxidized to the respective quinones by phenoloxidases such as laccase. Laccase 2, which is a key enzyme for the sclerotization and tanning pathway in beetles, has been already proposed to participate in the luciferin biosynthetic pathways^11,13,16,17,20,21^.

**Figure 2:**
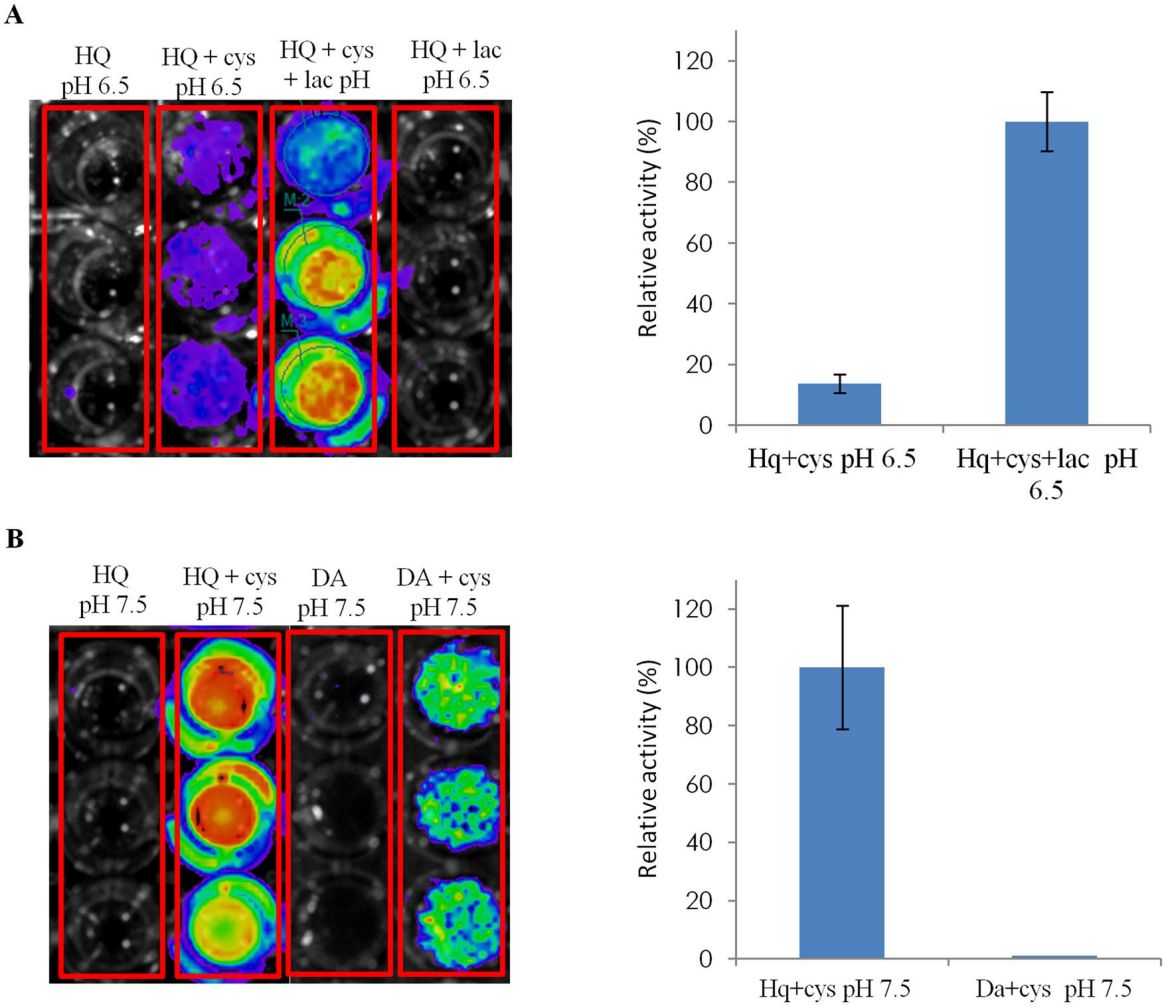
Bioluminescence CCD imaging obtained after mixing the reaction products of luciferin synthesis in the presence of *Amydetes vivianii* firefly luciferase and MgATP, and (right graphs) relative luminescent activity: (**A**) Reaction products of hydroquinone, cysteine, and laccase at pH 6.5, (**B**) Reaction products of hydroquinone and cysteine, and dopamine and cysteine at pH 7.5.

Thus, in order to investigate the involvement of hydroquinone and dopamine as precursors of luciferin biosynthesis, we performed spontaneous oxidation assays with hydroquinone and dopamine. At physiological pH (7.5) both hydroquinone and dopamine in the presence of cysteine spontaneously form luciferin, as can be observed by the bioluminescence produced in the assays where *Amydetes* firefly luciferase was mixed with MgATP (Fig. 2). At pH 7.5, the hydroquinone spontaneously oxidizes to benzoquinone, which then reacts with d-cysteine to produce d-luciferin, giving off intense bioluminescence upon mixing with firefly luciferase and MgATP. Dopamine also undergoes spontaneous oxidation to produce dopamine-quinone, which may also react with d-cysteine, but producing weaker bioluminescence, indicating that dopamine is less effective than benzoquinone to produce d-luciferin. At neutral pH, dopamine oxidation in the presence of cysteine is known to produce also the cysteinyldopamine and dihydrobenzothiazine intermediates^22,23^.

To investigate whether the enzyme laccase participates in formation of d-luciferin using hydroquinone as substrate, we also analyzed the bioluminescence produced after incubating laccase with hydroquinone and cysteine at pH 6.5, which is the optimum pH of commercial laccase with hydroquinone. The reaction of hydroquinone and d-cysteine in the presence of laccase produced considerably higher bioluminescence, than the control reaction in the absence of laccase, indicating the likely participation of laccase in luciferin biosynthesis from benzoquinones.

**Figure 3:**
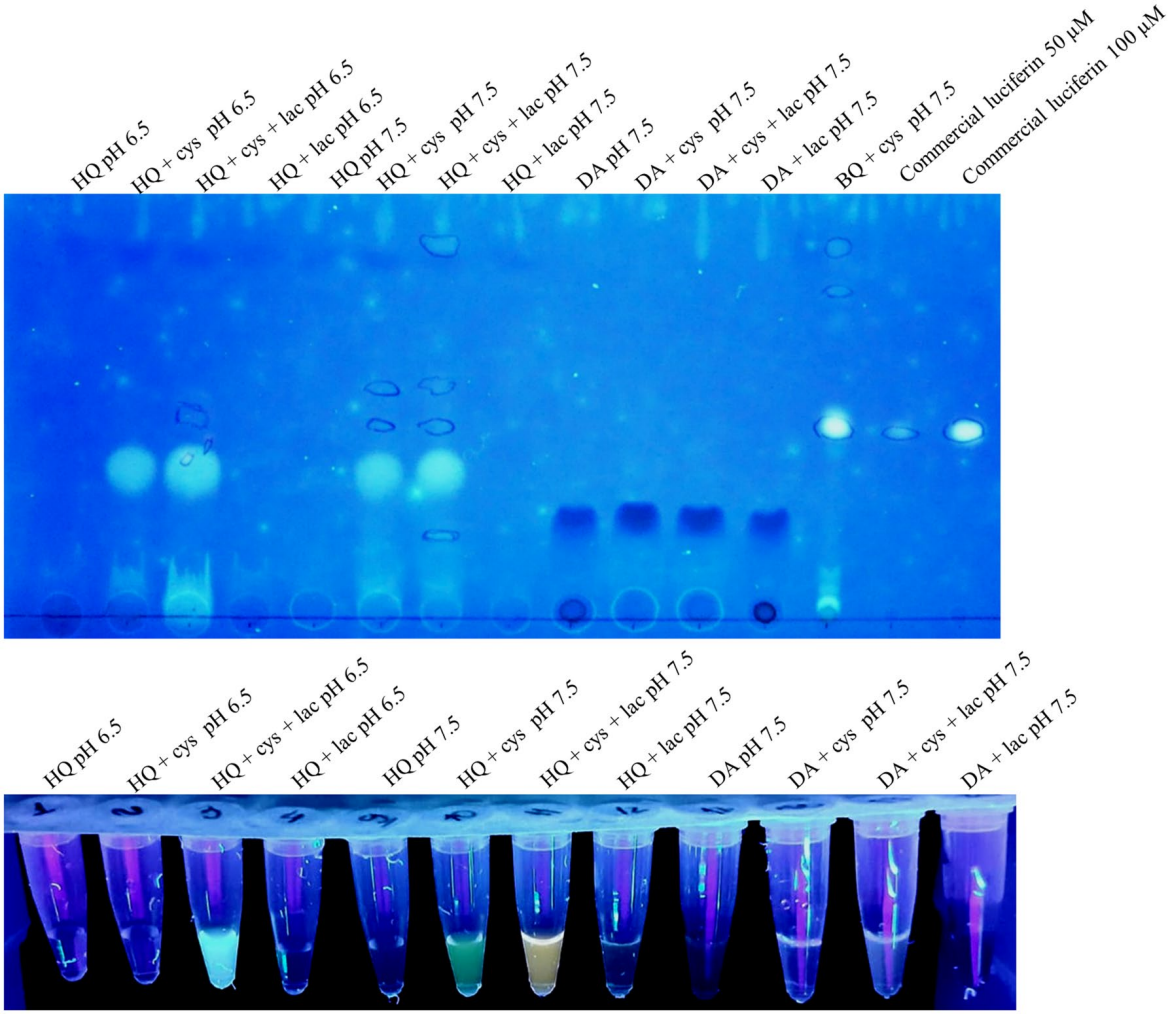
Thin Layer Chromatography of reactions of luciferin synthesis (upper panel) and fluorescence under UV light irradiation of these samples (lower panel).

The luciferin formation in these reactions of quinones and cysteine was also confirmed by TLC, fluorescence of the samples under UV light irradiation (Fig. 3), and bioluminescence spectra of the reaction products in the presence of *Amydetes vivianii* firefly luciferase and ATP (Supplementary Fig. S1). The bioluminescent spectra of *Amydetes* luciferase with the reaction products of hydroquinone + laccase + d-cysteine in buffer pH 6.5 displayed emission maxima of 551 nm, which is very close to that of this enzyme in presence of commercial firefly d-luciferin (547 nm) (Supplementary Fig. S1)^24,25^.

### Sensing in vivo luciferin synthesis in *E. coli* expressing firefly luciferase

In order to investigate whether luciferin synthesis occurs in bacteria, we prepared immobilized recombinant bacteria expressing *Amydetes vivianii* firefly luciferase. Bioluminescence emission by living bacteria expressing luciferase serves as an indicator of luciferin biosynthesis, as well as an indicator of cell viability, since bioluminescence can not be produced in the absence of the vital cofactor, ATP. The emission of bioluminescence by living bacteria upon treatment with *p*-benzoquinone and cysteine attests the luciferin formation inside the cells. In controls, where these compounds were added to bacteria independently, no light emission could be detected. We also tested these compounds alone or in combination on the cell viability assay through bioluminescence, upon addition of exogenous commercial d-luciferin.

**Figure 4:**
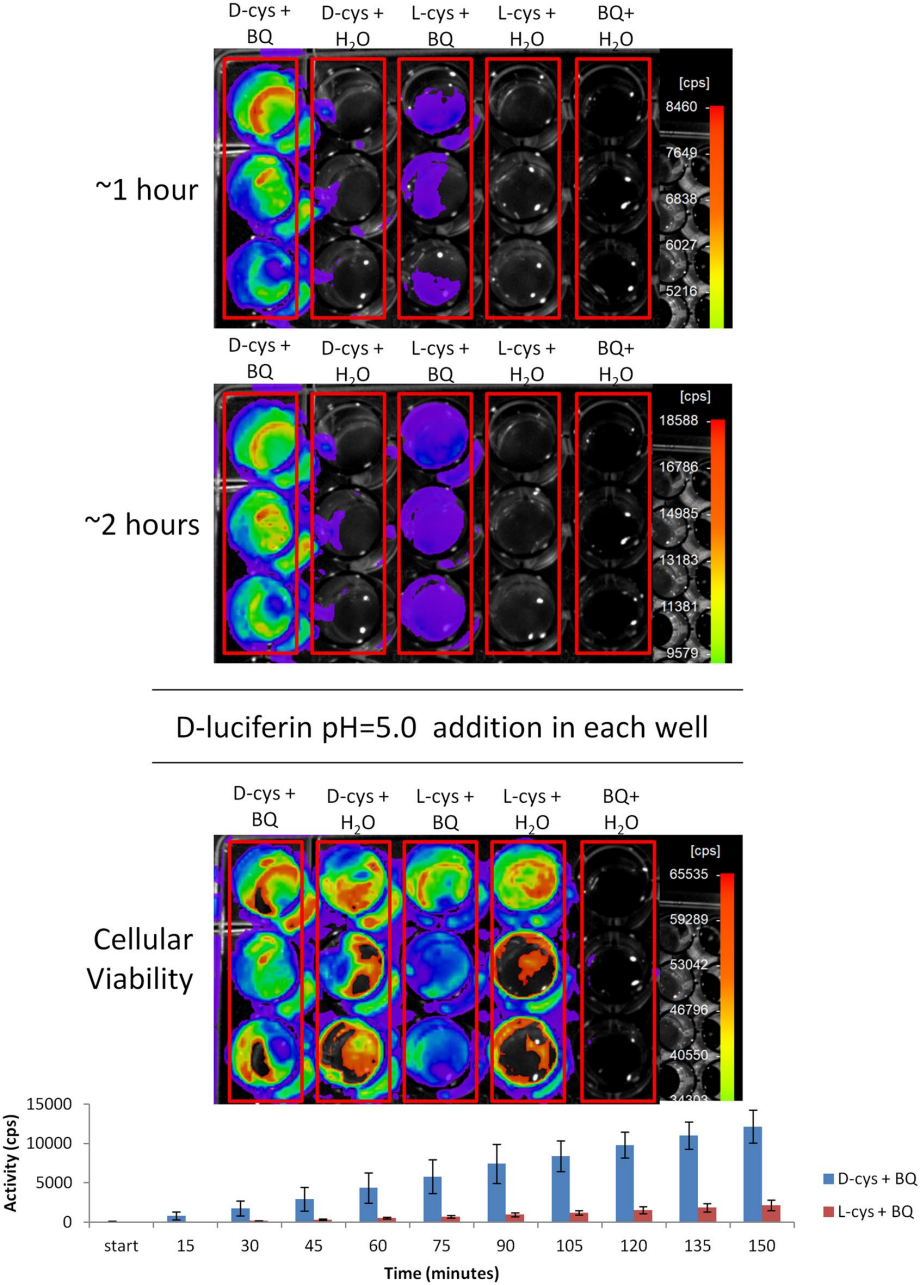
Bioluminescence of *E. coli* BL21 transformed with *Amydetes vivianii* firefly luciferase to sense luciferin formation from cysteine and *p*-benzoquinone. The cellular viability was analyzed by addition of 10 µL of 1 mM d-luciferin pH 5.0 in each well, at the end of the assay of luciferin formation. Bars graph shows the bioluminescent activity over time.

Figure 4 shows the bioluminescence of transformed bacteria in wells treated with: d-cysteine ​​and *p*-benzoquinone (d-cys + BQ) and with l-cysteine ​​and *p*-benzoquinone (l-cys + BQ). As expected, in the first case, the bioluminescence was very intense attesting d-luciferin formation. The enantioselectivity of beetle luciferases by the d-luciferin isomer for the bioluminescent reaction is already well known^3,7,11,12^. However, in the case of wells containing l-cysteine ​​and *p*-benzoquinone, a weaker bioluminescence could also be detected after ~ 30 min of incubation, indicating that a slower process of formation of d-luciferin is taking place in bacterial cells. This can be explained by the enantiomerization of l-luciferin into d-luciferin in the presence of luciferase, ATP, CoA and endogenous bacterial esterases, as previously demonstrated by Niwa et al.^12^.

Similar results were also obtained using *Pichia pastoris* yeast cells expressing *Pyrearinus termitilluminans* click beetle luciferase (Py), but with a weaker signal intensity (Supplementary Fig. S2).

### Benzoquinone toxicity and cysteine detoxification

After analyzing cell viability by bioluminescence, upon adding commercial d-luciferin, we noted that the wells treated only with *p*-benzoquinone did not display any bioluminescence, indicating toxic effect on the cells, as would be expected by the well known toxicity of benzoquinones. Benzoquinone and other quinones are very toxic to living cells, because they are very reactive toward nucleophilic groups such as amines of lysines, and thiols of cysteines of proteins, inactivating them, and also by generating reactive oxygen species, causing toxic effects to the cells^26^.

**Figure 5:**
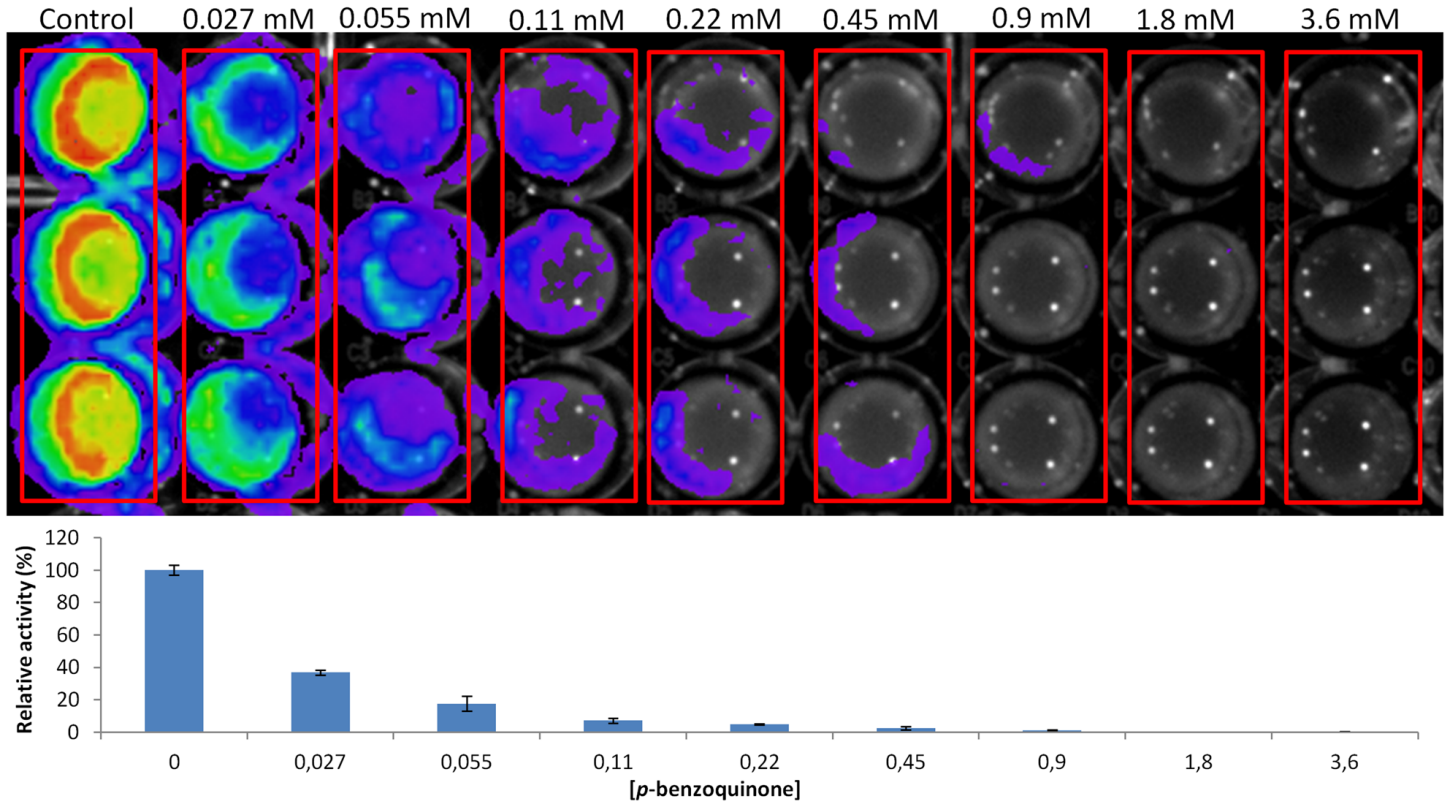
Toxic effect of *p-*benzoquinone at different concentrations on bacterial cell viability, analyzed by bioluminescence. The bars graph shows the bioluminescent activity at different *p*-benzoquinone concentrations.

To better evaluate the toxicity of benzoquinone in bacteria, a test was performed adding different concentrations of *p*-benzoquinone (0–3.6 mM) on immobilized bacteria expressing *Amydetes vivianii* firefly luciferase. We analyzed the cell viability after 2 h of exposure to *p*-benzoquinone. Figure 5 summarizes the results. At 0.22 mM, *p*-benzoquinone is already affecting cell viability, at 0.45 mM almost no bioluminescence is produced, and at higher concentrations the cells completely lost viability. Even at the lowest concentration of *p*-benzoquinone (0.027 mM), a loss of cell viability is already observed when compared to the control. The results indicate an I_C_50 (concentration that inhibits bioluminescent activity by 50%) of ~ 25 µM for *p*-benzoquinone. Similar results were observed in *Pichia pastoris* cells expressing *Pyrearinus termitilluminans* luciferase, but in this case the I_C_50 was ~ 100 µM for *p*-benzoquinone (Supplementary Fig. S3).

In the presence of cysteine, however, the cells remained viable even with a much higher concentration of *p*-benzoquinone (3.6 mM). Nucleophilic compounds, especially thiols, are highly reactive toward quinones^27^, forming less toxic adducts. Cysteine thiol groups spontaneously react with *p-*benzoquinone, decreasing its toxic effect and generating adducts such as cysteinyl DOPA and luciferin.

These results clearly indicate that, inside cells, free cysteine ​​may naturally act in a benzoquinone detoxification process, competing with the thiols and other nucleophilic groups of proteins, with the concomitant formation of d-luciferin.

### Firefly luciferin synthesis is related to the sclerotization/tanning pathways

**Figure 6:**
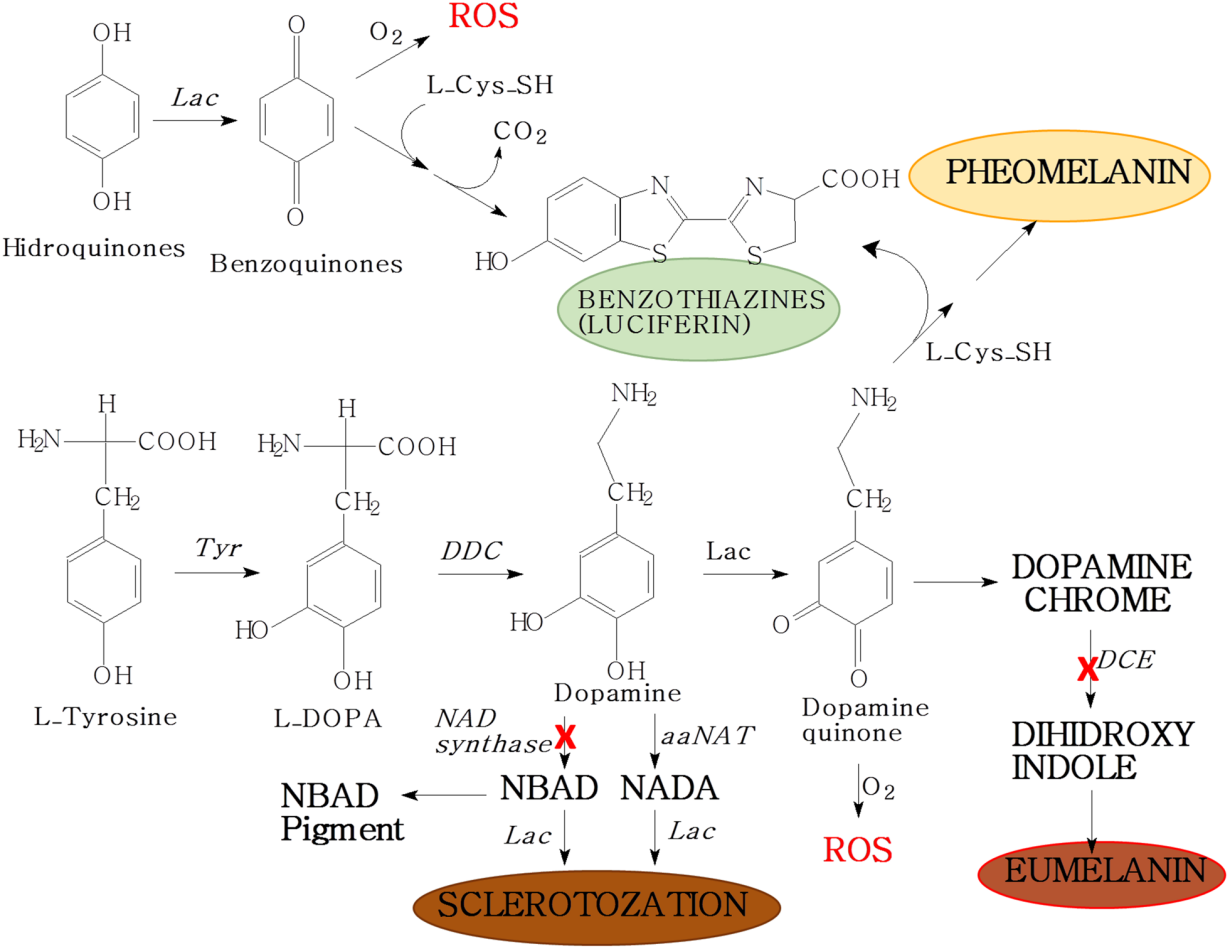
A proposed luciferin biosynthetic pathway as an alternative cysteine-dependent quinone detoxification route departing from the sclerotization and melanization pathways in beetles. The red crosses indicate enzyme deficient pathways that may lead to quinone accumulation and toxicity. *Tyr* Tyrosinases, *Lac* Lacases, *DDC* dopa decarboxylase, *aaNAT* dopamine *N*-acetil transferase, *DCE* dopachrome isomerase.

In nature, benzothiazolic compounds are found in melanins, thiamin, and antibiotics. Because thiamin and antibiotics are not usually synthesized by higher eukaryotes, it is more likely that these compounds in insects arise from melanogenesis, or are acquired from the diet^21^. Melanogenesis involves two main branches, according to the nucleophile which reacts with quinones, eumelanogenesis and pheomelanogenesis (Fig. 6). Eumalonogenesis, which uses amines as nucleophilic groups, is responsible for formation of darker eumelanin. On the other hand, pheomelanogenesis uses cysteine to produce the benzothiazolic pheomelanin^28^.

The luciferin biosynthetic pathway involves cysteine and benzoquinones, which are also precursors involved in the cuticle sclerotization and melanization pathways. Quinones from different origins, such as hydroquinone stored as arbutin glycoside^13^, benzoquinone acetic acid from tyrosine degradation^20^, or quinones intermediates of pigmentation/sclerotization pathway such as DOPA-quinone^21^, in the presence of cysteine, produce benzothiazothiazines, such as firefly luciferin. Therefore, it is possible that there is a common link between these metabolic pathways (Fig. 6).

Genomic and transcriptomic analysis indeed showed the presence of gene products involved with pigmentation/sclerotization pathway in firefly lanterns^16–20^. This is in part expected because the lanterns also involve their cuticles. To better understand the possible involvement of transcripts related to quinone and sclerotization/tanning pathways with luciferin synthesis, we reviewed our previous transcriptomic data from firefly lanterns and non-bioluminescent tissues in beetles^18,19,29,30^.

Melanin synthesis and sclerotization in beetles depart from the tyrosine metabolism, involving several common precursors, including quinone intermediates (Fig. 6). DOPA is produced from tyrosine hydroxylation by the enzyme tyrosine hydroxylase, and DOPA decarboxylase produces dopamine from DOPA. Phenoloxidases, such as laccases, catalyze DOPA and dopamine (preferential substrate for phenoloxidase) oxidation to the corresponding quinones.These quinones are converted into dopachrome or dopamine-chrome and then to 5,6-dihydroxyndole by dopachrome isomerase, which is finally oxidazid and polymerized producing melanin-like pigments^31–34^.

Quinones are highly reactive agents toward protein nucleophilic groups such as amines of lysines, imidazole imine groups from histidines and thiols of cysteines, during cuticle hardening or sclerotization^32^. Dopamine can also be *N*-acetylated to *N-*acetyldopamine (NADA) by *N*-acetyltransferase enzyme or to *N*-β-alanyldopamine (NBAD) by NBAD synthetase, and these catecholamines can be finally oxidized to their corresponding quinones by phenoloxidases. NADA-quinones and NBAD-quinones are then used in pigmentation (NADA pigment and NBAD pigment) and sclerotization^32,34^.

Tyrosine hydroxylase, DOPA-decarboxylase (DDC), laccase 2 (Lac), and dopamine *N*-acetyl transferase were found in the transcriptomes of all the analyzed photogenic and non-photogenic tissues. However, their abundances, analyzed by the abundance values in FPKM (Fragments Per Kilobase Million), are comparatively lower in both bioluminescent and non-bioluminescent tissues when compared to the most abundant transcripts in each tissue (Table 1).

**Table 1:** Transcripts similar to enzymes involved with pigmentation/sclerotization pathway that were found in firefly lanterns and non-bioluminescent tissues of Coleoptera species. Abundance values (FPKM) of these transcripts also are exhibited.

Specie	Tissue	FPKM value of the most abundant transcript in the tissue	Tyrosine hydroxylase abundance (FPKM)	Dopa decarboxylase abundance (FPKM)	Dopamine *N*-acetyl transferase abundance (FPKM)	Laccase 2 abundance (FPKM)
*A. lineatum*	Lantern (larvae)	36,226.08	21.17	Absent	13.53	1.18
	Lantern (adult)	13,142.3	1768.49	2.09	6.23	86.41
	Fat body (larvae)	88,043.62	1.54	1.09	18.62	14.34
*P. fragilis*	Lantern	34,216.12	14.35	51.38	12.15	3.84
	Fat body	18,512.04	1.90	20.45	7.01	Absent
*P. hirtus*	Lateral lantern	47,492.39	Absent	43.61	7.17	5.25
	Fat body	14,648	10.14	50.37	Absent	19.08
*Z. morio*	Fat body (larvae)	52,837.3	58.15	39.06	2.85	Absent
	Malpighian tubules (larvae)	92,736.94	2.26	2.92	80.34	16.08
*C. opacus*	Abdomen	24,747.26	21.24	15.41	38.86	20.55

Deficiency or expression alterations of some of these enzymes are known to cause changes in the pigmentation patterns and structure of the cuticle^35–37^. Loss of laccase 2 and dopachrome isomerase (DCE) in *Tenebrio molitor* (Coleoptera), for example, resulted in pale/white and light yellow/brown cuticle, respectively^37^. Mutants deficient in NBAD synthetase exhibit softer and darker cuticle. This phenotype is explained because the NBAD is not produced and the resulting excess dopamine is therefore driven to synthesis of the dark pigment^36^.

In the case of firefly lanterns, despite showing several transcripts putatively involved in the production of dopamine quinone and NADA quinone from dopamine and NADA, which are important intermediates for pigmentation/sclerotization, no transcripts similar to dopachrome isomerase (DCE) nor NBAD synthetase enzyme were found. The absence of dopachrome isomerase in these tissues suggest that dopamine is not converted to eumelanins, whereas the absence of NBAD synthetase indicate that NBAD and its quinone product are not produced in the photogenic and non-photogenic tissues of luminescent beetles. In the absence of NBAD, the equilibrium may therefore shift toward dopamine and dopamine-quinone accumulation, causing potential cytotoxic effects, which include enzymes inactivation and oxidative stress by generated ROS, and/or favoring melanization pathways (Fig. 6).

Altogether, the presence of laccase 2 and dopamine *N*-acetyl transferase, and the absence of dopachrome isomerase and NBAD synthetase in firefly lanterns may explain the lighter coloration and softer cuticle found in the lantern regions, in agreement with our hypothesis that the primitive lanterns evolved in the more translucent and less sclerotized areas in larval fireflies^38^.

Furthermore, the higher abundance of enzymes related to cysteine metabolism in the photogenic tissues, which include cystathionine-β-synthase (CBS), that converts homocysteine to cystathionine, and cystathionine-β-lyase (CBL), that converts cystathionine to l-cysteine, indicate that the photogenic tissues produce high levels of cysteine, favoring the pheomelanogenesis pathway and production of benzothiazines, including luciferin^16,18–20,29^. On the other hand, in other tissues of beetles such as the Malpighian tubules and the fat body of the non-bioluminescent *Z. morio* larvae, these enzymes exhibit low abundance.

### Luciferin as a by-product of a quinone detoxification pathway

Altogether, the lack of enzymes involved in final steps of the sclerotization pathway, the highly active cysteine metabolism in the photogenic tissues, and the evident role of cysteine in detoxification of quinones, indicate that luciferin may have first appeared during the evolution of beetles as a byproduct of cysteine-dependent detoxification of accumulated quinone intermediates in a deficient sclerotization pathway (Fig. 6). It is not surprising that in most extant Elateroidea beetle families, bioluminescence is found mainly in larvae and adults of the soft-bodied Phengodidae, Ragophtalmidae and Lampyridae families (former Cantharoidea superfamily), which display a weaker sclerotization. The exceptions are the hard-bodied Elateridae adults, in which BL appears in larvae which are in general less sclerotized, and in lanterns under less-sclerotized and pigmented cuticles of the prothorax and in hidden lanterns under the strongly sclerotized areas of the abdomen which are exposed only during flight (abdominal lanterns).

## Concluding remarks

Here we showed that *E. coli* bacteria expressing firefly luciferase can synthesize firefly d-luciferin using *p*-benzoquinone and d-cysteine and, to a lesser extent, also with l-cysteine which is gradually converted into d-luciferin, in the presence of endogenous luciferase, CoA and bacterial esterases. Laccase is shown to participate in the biosynthetic pathway of luciferin by oxidizing hydroquinone to benzoquinone. Noteworthy, the production of luciferin in the presence of cysteine considerably decreases the natural toxicity of *p*-benzoquinone to bacterial cells. This result, the highly active cysteine metabolism in firefly lanterns, and the lack of terminal enzymes involved in the sclerotization pathway in the transcriptomes of photogenic tissues of fireflies, suggest for the first time the possibility that luciferin may have arosen as a byproduct of an alternative cysteine-mediated detoxification route of accumulated quinones during early days of sclerotization/tanning in beetles.

## Methods

### Luciferase expression and purification

For *A. vivianii* luciferase expression, transformed *E. coli* BL21-DE3 cells were grown in 100 mL of LB medium at 37 °C up to OD_600_ = 0.4, and then induced at 18 °C with 0.4 mM IPTG during 18 h. Cells were harvested by centrifugation at 2.500×*g* for 15 min and resuspended in extraction buffer consisting of 50 mM sodium phosphate buffer, pH 7.0, 300 mM NaCl, 10 mM imidazole, and protease inhibitor cocktail (Roche), lysed by ultrasonication and centrifuged at 15.000×*g* for 15 min at 4 °C. The N-terminal histidine-tagged Amy luciferase were further purified by agarose-Nickel affinity chromatography (QIAGEN), using the following buffers: (Wash buffer) 50 mM Phosphate pH 7.0, 300 mM NaCl, 20 mM Imidazole; (Elution buffer) 50 mM Phosphate pH 7.0, 300 mM NaCl, 250 mM Imidazole and (dialysis buffer) 25 mM Tris–HCl pH 8.0, 10 mM NaCl, 1 mM EDTA, 2 mM DTT, and 10% glycerol.

### Luciferin spontaneous synthesis

The non-enzymatic synthesis of luciferin from cysteine and *p-*benzoquinone (1:1), reported by Kanie et al. was replicated in this study using purified *A. vivianii* firefly luciferase (AmyLuc) and 4 mM ATP in 90 mM Tris–HCl pH 7.5 buffer and 5 mM MgSO4 to sense the formation of D-luciferin. For this purpose, reactions different compounds were mixed and analyzed in an Elisa plate. The following treatments were tested: (1) Buffer + MgATP; (2) Buffer + MgATP + AmyLuc; (3) Buffer + MgATP + AmyLuc + d-cys + BQ; (4) Buffer + MgATP + AmyLuc + d-Cys; (5) Buffer + MgATP + AmyLuc + l-cys + BQ; (6) Buffer + MgATP + AmyLuc + l-cys; (7) Buffer + MgATP + AmyLuc + l-cys; (8) Buffer + MgATP + AmyLuc + d-cys + Hydroquinone; (9) Buffer + MgATP + AmyLuc + l-cys + Hydroquinone; (10) Buffer + MgATP + AmyLuc + Hydroquinone. The luciferin formation was followed by bioluminescence using Berthold Nightowl CCD camera with images taken every 15 min, and also confirmed by detection of fluorescence upon irradiation by UV light on the Elisa plate. The assays were performed in triplicate.

### Thin layer chromatography (TLC)

To confirm the presence of luciferin in the reaction, thin layer chromatography (TLC) was also performed. For this purpose, commercial luciferin (Promega) was used as a standard for comparison with the luciferin obtained from the reaction of *p*-benzoquinone and cysteine. On a silica gel plate (Merck), 1 μL of 0.1 mM d-luciferin, 1 μL of the mixtures of benzoquinone with l-cysteine or d-cysteine were spotted. The mobile phase was ethyl acetate; ethanol and H_2_O (5: 2: 3). After chromatography, the fluorescent bands were visualized upon irradiation with UV light and the migration coefficients of the samples were calculated.

### Immobilization of bioluminescent bacteria

For bioluminescent bacteria immobilization, 1 mL of *E. coli* transformed with pCold-Amy vector expressing the luciferase at OD_600_ ~ 1.0, and 9 ml of Top agar (LB broth with 0.7% [w/v] agarose) with ampicillin were mixed at 42 °C in a sterile Falcon tube, and 100 μL of this mixture were quickly transferred in to each well of the 96-well microplate. The plate was left standing during 24 h at 22 °C before the assays.

### In vivo assay of luciferin synthesis in bacteria

The formation of luciferin in bacteria was monitored by bioluminescence using the immobilized *E. coli* expressing *A. vivianii* firefly luciferase (as described above), in the presence of cysteine and benzoquinone precursors. The immobilized bacteria inside the wells were treated with following assay mixtures: (1) 5 μL of 80 mM *p*-benzoquinone + 5 μL of H_2_O; (2) 5 μL of 80 mM d-cysteine + 5 μL of H_2_O; (3) 5 μL of L-cysteine + 5 μL of H_2_O; (4) 5 μL of 80 mM *p*-benzoquinone + 5 μL 80 mM d-cysteine; (5) 5 μL of 80 mM *p*-benzoquinone + 5 μL of 80 mM l-cysteine. All treatments were performed in triplicate. The bioluminescence was detected using a NightOwl CCD camera system (Berthold). At the end of the experiment, 10 μL of 1 mM d-luciferin pH 5.0 were pipetted into each well, to analyze the cell viability by bioluminescence as an indicator of ATP. The assays were performed in triplicate.

### Assay of *p*-benzoquinone toxicity in cell viability

Knowing that *p*-benzoquinone at a concentration of 3.6 mM is very toxic to immobilized bacteria, we then tested different concentrations of this reagent to avail cell viability. Bacteria immobilized on an Elisa plate were exposed for 2 h at 22 °C to the following *p*-benzoquinone concentrations: 0.027 mM, 0.05 mM, 0.11 mM, 0.22 mM, 0.45 mM, 0.9 mM, 1.8 mM and 3.6 mM. After this period, 10 µL of 1 mM luciferin pH 5.0 were added into each well and the bioluminescence production analyzed in a CCD photodetection camera, as an indicator of the presence of ATP and therefore of cell viability. The obtained images were analyzed by densitometry using the program IndiGO, to estimate the I_C_50 (Concentration of a given compound which causes 50% inhibition of bioluminescent activity). The I_C_50 was calculated according to Quest Graph™ IC50 Calculator^39^.

### In vitro luciferin synthesis using laccase enzyme

The participation of laccase in luciferin enzymatic synthesis was assayed using commercial laccase from *Rhus vernicifera* (Sigma, USA) in the presence of hydroquinone (HQ) or dopamine (DA), and d-cysteine. The reactions were initially performed by mixing 10 μL of laccase (0.5 U) and 25 μL of 40 mM HQ or DA, in a final volume of 100 μL of 0.10 M phosphate buffer at pH 6.5, or 90 mM Tris–HCl buffer pH 7.5 in the Elisa plate wells followed by incubation during 1 h at 22 °C under moderate agitation. After that, 12.5 μL of 80 mM d-cysteine were added to the wells with further incubation during 18 h. The control reactions were performed in the absence of d-cysteine or laccase. Luciferin synthesis in each reaction was luminometrically evaluated by bioluminescence in the presence of Amy luciferase and ATP. This assay was carried out by mixing 25 μL of the reaction product, 5 μL of Amy luciferase (~ 3 µg), 5 μL of solution containing 80 mM MgSO_4_/40 mM ATP, and 65 μL of 0.10 M Tris–HCl buffer at pH 8.0. The reactions were performed in triplicate, and the bioluminescent activities were normalized in relation to the activity of the control reaction between hydroquinone and cysteine at pH 7.5. In order to check luciferin formation, we also carried out TLC of these samples, as described in the topic 2.3.

### Bioluminescence images analysis and densitography

Bioluminescence imaging was done using a NightOwl II CCD camera system after 2 min of exposition (Berthold, Germany), and the obtained images were analyzed by densitometry using the program IndiGO.

### Bioluminescence spectra

The bioluminescence spectra of the luciferin synthesis reaction in the presence of *Amydetes* firefly luciferase were measured using an ATTO model AB-18505 spectroluminometer (Tokyo, Japan). For this assay, 25 μL of reaction product were mixed with 5 μL of Amy luciferase, 5 μL of solution containing 80 mM MgSO_4_/40 mM ATP, and 65 μL of 0.10 M Tris–HCl buffer at pH 8.0. The spectra were scanned from 400 to 700 nm.

### Transcriptional analysis

Sequencing, assembly and first analysis of bioluminescent and non-bioluminescent beetle transcriptomes were previously published by our research group^18,19,29,30^. In this work, we re-analyzed transcriptomic data from firefly lanterns and non-bioluminescent tissues of Coleoptera species to search for transcripts related to insect cysteine, quinone, and pigmentation/sclerotization pathways to find possible links with luciferin synthesis.

## Supplementary Information

Supplementary Information.

## Data Availability

The SRA (Sequence Raw Reads) analyzed in this study are deposited on NCBI database (project number: PRJNA475874, PRJNA347807, PRHNA347512, PRJNA400859).
