# Supplementary material for: Biosensing firefly luciferin synthesis in bacteria reveals a cysteine-dependent quinone detoxification route in Coleoptera
Source: Sci Rep. 2022 Aug 31;12:14815. doi: 10.1038/s41598-022-17205-z (PMC9433453; doi:10.1038/s41598-022-17205-z)
Supplement: Supplementary file 1 — Supplementary Information. [file 41598_2022_17205_MOESM1_ESM.docx]

**Supplementary Information**

**Biosensing firefly luciferin synthesis in bacteria reveals a cysteine-dependent quinone detoxification route in Coleoptera**

 Daniel Rangel de Souza^1^, Jaqueline Rodrigues Silva^2^, Ariele Moreira^2^, Vadim Viviani^1,2*^

^1^ Graduate Program of Biotechnology and Environmental Monitoring, Federal University of São Carlos, Brazil.

^2^ Departament of Physics, Chemistry and Mathematics, Federal University of São Carlos, Brazil.

*email: [viviani@ufscar.br](mailto:viviani@ufscar.br)

**Supplementary Figures**


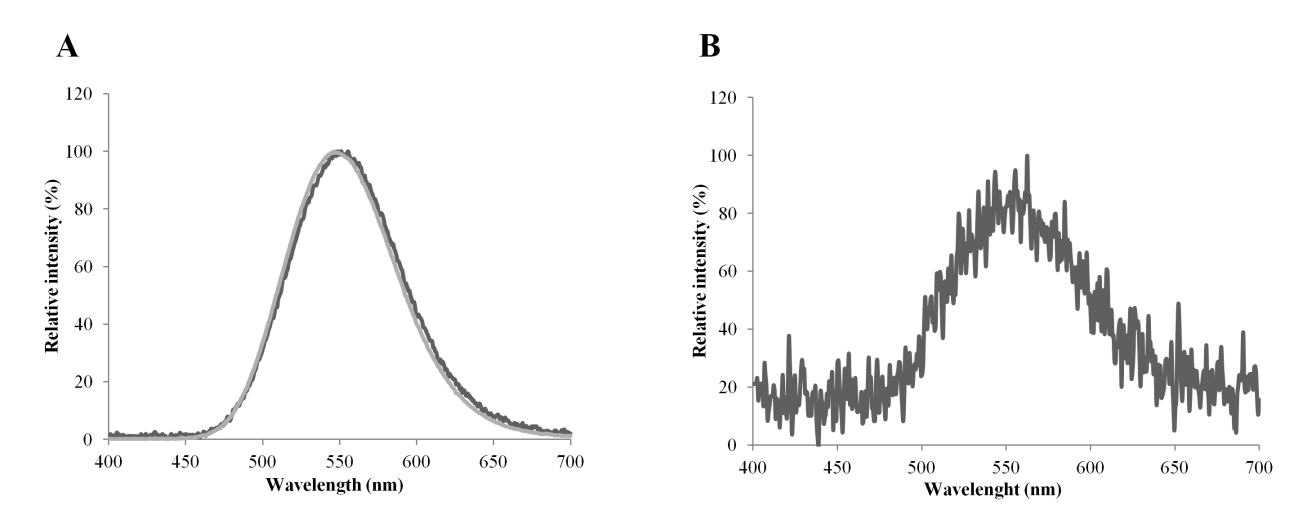


**Supplemenatary figure S1:** Bioluminescence spectra of luciferin synthesis reactions in presence of *Amydetes* firefly luciferase and MgATP. Panel A: overlay of normalized bioluminescence spectra of Amy luciferase in presence of D-luciferin (grey) and Amy luciferase in the presence of the reaction products of hydroquinone + laccase + D-cysteine in buffer at pH 6.5 (black). Panel B: bioluminescence spectra of reaction of hydroquinone + D-cysteine in buffer at pH 7.5.


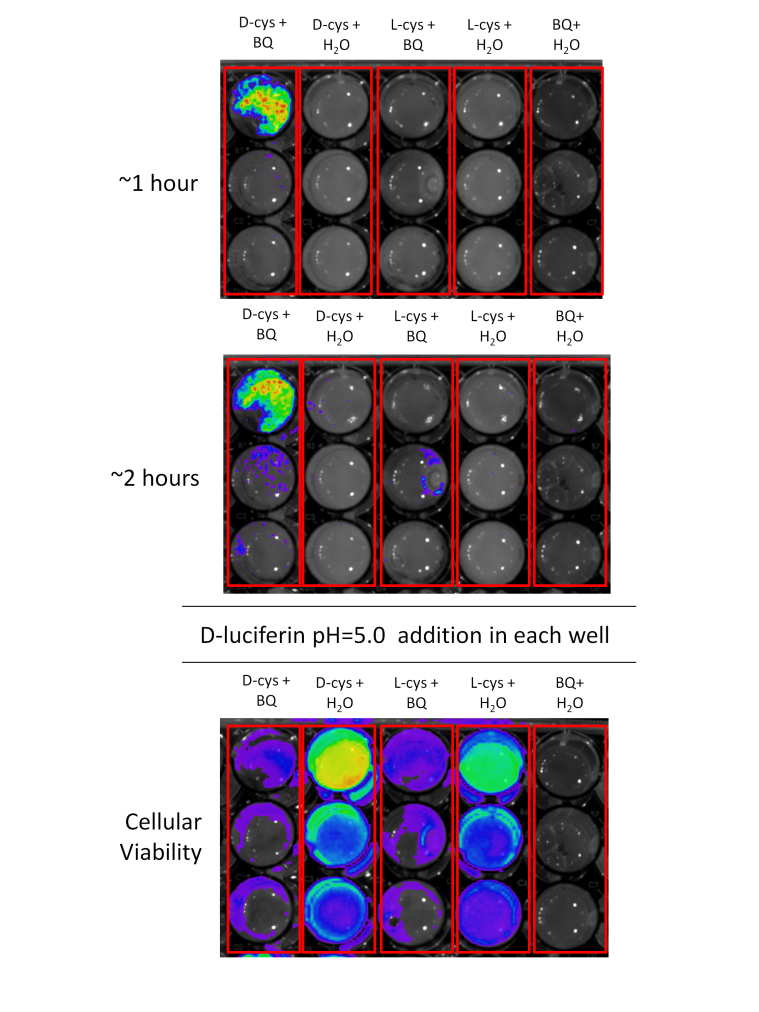


**Supplementary figure S2.** Bioluminescence of *Pichia pastoris* cells transfected with *Pyrearinus termitilluminans* luciferase to sense luciferin formation from cysteine and *p*-benzoquinone. The cellular viability was analyzed by addition of 30 µL of 1 mM D-luciferin pH 5.0 in each well, at the end of the assay of luciferin formation.

**
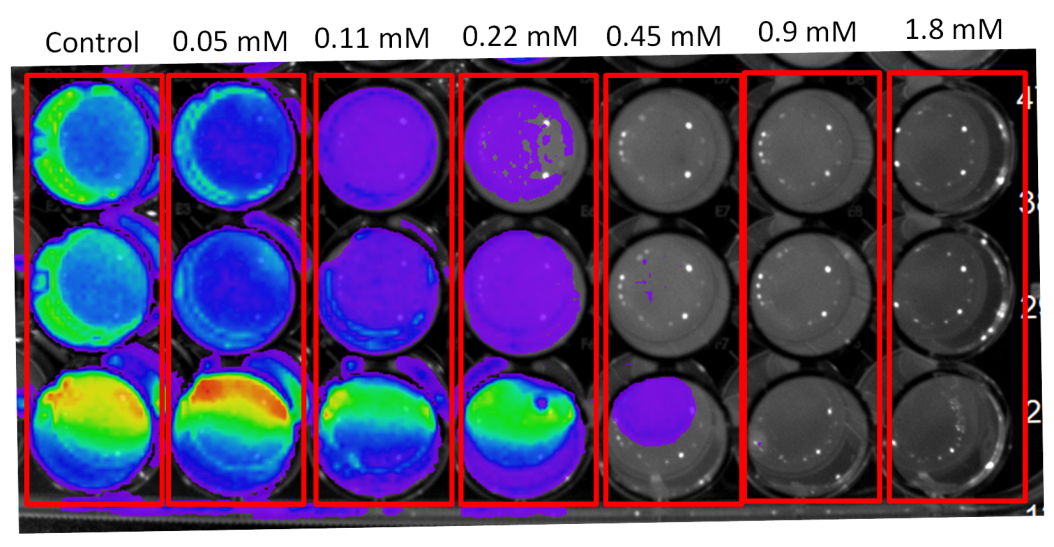
**

**Supplementary figure S3.** Inhibition of *Pichia pastoris* cells bioluminescence by different concentrations of *p-*benzoquinone.
